# Commentary on: Pedraza N, Marciano MA. Simple and nondestructive method to access and process material on coverslipped slides. J Forensic Sci. 2025;70(5):2006–9. https://doi.org/10.1111/1556‐4029.70083

**DOI:** 10.1111/1556-4029.70448

**Published:** 2026-08-20

**Authors:** Natalia Pedraza, Michael A. Marciano

**Affiliations:** ^1^ Forensic & National Security Sciences Institute Syracuse University Syracuse New York USA

See Original Article *here*

Editor,

We write to provide an update to our previously published method for accessing and processing material on coverslipped slides [1].

Following publication, we were contacted by two accredited laboratories: a US‐based state laboratory and an international regional laboratory. The state laboratory asked whether our removal method could recover coverslips and hairs embedded in Permount (Fisher Scientific), a mounting medium not evaluated in our original study but commonly encountered in older casework. To address this question, we evaluated our humidity‐based method in a mini‐study using two microscope slides mounted with Permount and found it effective, permitting recovery of fully intact hairs from the slide surface. Coverslip removal required approximately 1 week in the humidity chamber; following removal, hairs could not be recovered until the slide and coverslip (depending on which surface the hair was bound to) were returned to the humidifier for an additional 24–48 h to further loosen the mounting medium and assist recovery. The condition of the recovered hairs was variable: some retained mounting medium as a hardened “crust,” while others showed little to no visible residue. Notably, the recovered hairs were brittle and prone to breakage when handled with tweezers.

In a second mini‐study, the international laboratory noted above evaluated our method on five microscope slides prepared from the sperm fractions following differential extraction and mounted with Biomount‐BT, a d‐limonene‐based medium (Daniels Health). The laboratory required access to the sperm cells for subsequent profiling. All five attempts to remove the coverslip were successful.

Although the sample sizes in these mini‐studies were small, coverslip removal succeeded in all slides attempted (2 of 2 and 5 of 5, respectively), and we anticipate the method will continue to perform reliably across casework applications, with older slides potentially requiring longer incubation in the humidity chamber. These findings suggest that our method is broadly applicable across mounting media of varying composition and age and has meaningful utility in trace evidence analysis, where physical evidence can be accessed and recovered nondestructively. This expands the applicability of our method to a wider range of forensic casework and materials, offering new opportunities for the reexamination of archival slides and other coverslipped evidence. Because forensic practitioners rely on published methods rather than informal or anecdotal reports when making casework decisions, both our group and the contacting laboratories felt these findings should be disseminated through a journal for the benefit of the broader forensic science community.

## FUNDING INFORMATION

This study was funded by Syracuse University Syracuse Office of Undergraduate Research & Creative Engagement (SOURCE) and the Forensic and National Security Sciences Institute.

## CONFLICT OF INTEREST STATEMENT

The authors have no conflicts of interest to report.

## CONSENT

This study did not involve the use of human subjects' research.
